# Comparative in vitro activity of Delafloxacin and other antimicrobials against isolates from patients with acute bacterial skin, skin-structure infection and osteomyelitis

**DOI:** 10.1016/j.bjid.2024.103867

**Published:** 2024-09-18

**Authors:** Ághata Cardoso da Silva Ribeiro, Fernanda Fernandes Santos, Tiago Barcelos Valiatti, Michael Henrique Lenzi, Ingrid Nayara Marcelino Santos, Raíssa Fidelis Baêta Neves, Ikechukwu Benjamin Moses, Jaqueline Pilon de Meneses, Renata Gebara de Grande Di Sessa, Mauro José Salles, Ana Cristina Gales

**Affiliations:** a Universidade Federal de São Paulo (UNIFESP), Escola Paulista de Medicinan (EPM), Departamento de Medicina Interna, Divisão de Doenças Infecciosas, Laboratório Alerta, São Paulo, SP, Brazil; b Ebonyi State University, Faculty of Science, Department of Applied Microbiology, Abakaliki, Nigeria; c Eurofarma, Brazil

**Keywords:** Acute bacterial skin, Skin-structure infection, Osteomyelitis, Delafloxacin, Antimicrobial-resistant bacteria

## Abstract

The aim of this study was to compare the in vitro activity of delafloxacin with other fluoroquinolones against bacterial pathogens recovered from inpatients with osteomyelitis, Acute Bacterial Skin and Skin-Structure Infections (ABSSSI). In total, 100 bacterial isolates (58 % Gram-negative and 42 % Gram-positive) recovered from inpatients between January and April 2021, were reidentified at species level by MALDI-TOF MS. Antimicrobial susceptibility testing was conducted using the broth microdilution method and the detection of biofilm formation was assessed through the microtiter plate assay. The screening for *mecA* was carried out by PCR, while mutations in the Quinolone Resistance Determining Regions (QRDR), specifically *gyrA* and *parC*, were analyzed using PCR followed by Sanger sequencing. Results showed that delafloxacin exhibited greater in vitro potency (at least 64-times) than the other tested fluoroquinolones (levofloxacin and ciprofloxacin) when evaluating *Staphylococcus aureus* (MIC_50_ ≤0.008 mg/L) and coagulase-negative *Staphylococcus* (MIC_50_ 0.06 mg/L). Furthermore, delafloxacin (MIC_50_ 0.25 mg/L) was at least 4 times more potent than other tested fluoroquinolones (MIC_50_ 1 mg/L) against *P. aeruginosa*. No difference in delafloxacin activity (MIC_50_ 0.03 mg/L) was observed against *Enterobacter cloacae* when compared with ciprofloxacin (MIC_50_ 0.03 mg/L). Despite presenting low activity against *K. pneumoniae* isolates (22.2 %), delafloxacin exhibited twice the activity compared to both levofloxacin and ciprofloxacin. Delafloxacin also exhibited a strong activity (71.4 %‒85.7 %.) against biofilm producing bacterial pathogens tested in this study. Interestingly, 82.14 % of the staphylococci tested in this study harbored *mecA* gene. In addition, the *gyrA* and *parC* genes in fluoroquinolone-resistant Gram-negative isolates displayed different mutations (substitutions and deletions). Herein, we showed that delafloxacin was the most active fluoroquinolone against staphylococci (including MRSA) and *P. aeruginosa* when compared to other fluoroquinolones such as ciprofloxacin and levofloxacin.

## Introduction

Antimicrobial resistance is one of the main threats to human health. In the last years, the rates of Multidrug-Resistant (MDR) bacteria have increased; thus, limiting treatment options which have encouraged the development of new antimicrobials.^1^^,^^2^ In this sense, recently, a new fluoroquinolone, delafloxacin, was developed and approved by Food and Drug Administration (FDA) and European Medicines Agency (EMA) to treat Acute Bacterial Skin and Skin-Structure Infections (ABSSSI),^2^ and lately it has also been approved in the USA for the treatment of community-acquired pneumonia^3^ and, more recently, in Brazil launched in 2022 also for treatment of ABSSSI.

Delafloxacin presents an anionic nature which provides improved activity in the infection site. During the infectious process, the environment tends to become acidic (excess of free protons), and unlike other fluoroquinolones, delafloxacin undergoes protonation within this environment, turning into a neutral molecule that can easily enter the bacterial cell. Once inside the bacteria (neutral pH), delafloxacin deprotonates and initiates its mechanism of action.^4^^,^^5^ Delafloxacin is a bactericide broad-spectrum anionic fluoroquinolone that targets both bacterial DNA gyrase and topoisomerase IV, enzymes of Gram-positive and Gram-negative bacteria.^5^, ^6^, ^7^, ^8^

Regarding its use in clinical practice, delafloxacin has the advantage of being administered Intravenously (IV) (300 mg) and orally (450 mg) every 12 h. The Oral Administration (OR) shows a comparable bioavailability with IV, allowing the transition of therapy from IV to OR, and thus facilitating patient discharge.^9^^,^^10^ However, in Brazil, only the IV presentation is available.^11^

Recent studies have shown the efficacy of delafloxacin against both Methicillin-Susceptible *Staphylococcus aureus* (MSSA) and Methicillin-Resistant (MRSA), achieving up to 97.5 % of MRSA susceptibility. Moreover, it was observed that delafloxacin showed good activity against *Pseudomonas aeruginosa*.^12^, ^13^, ^14^

The present study aimed to evaluate the activity of delafloxacin in comparison to other antimicrobial agents against isolates recovered from patients diagnosed with ABSSSI or osteomyelitis in a tertiary hospital from the city of São Paulo, Brazil.

## Material and methods

### Bacterial isolates

A total of 100 isolates recovered from patients diagnosed with ABSSSI or osteomyelitis were collected between January and April 2021. The isolates identification at species level was performed by Matrix Assisted Laser Desorption Ionization ‒ Time of Flight Mass Spectrometry (MALDI-TOF MS) using the Microflex spectrometer LT (Bruker Daltonics, Massachusetts, USA). The data obtained was analyzed by Biotyper version 3.1 software (Bruker Daltonics, Massachusetts, USA). Scores ≥ 2.0 to 2.99 were considered trustful for species-level identification, while scores ≥ 1.7 to 1.99 were considered sufficient for genus-level identification.^15^

### Antimicrobial susceptibility testing

The antimicrobial susceptibility profile of the isolates was determined by broth microdilution method.^16^ The antimicrobials tested for each species were those recommended (Table 1). Quality control and the interpretation of results were performed according to BrCAST/EUCAST guidelines, with results following within the expected ranges. Since the FDA provides a broad range of delafloxacin MIC (Minimum Inhibitory Concentration) for different species, these FDA breakpoints were used to categorize the MICs of delafloxacin. Also, we used the delafloxacin breakpoints for *S. haemolyticus* to categorize other CoNS (Coagulase-Negative Staphylococci). The quality control strains used in this study were *Escherichia coli* ATCC 25922, *Pseudomonas aeruginosa* ATCC 27853, and *Staphylococcus aureus* ATCC 29213.^16^

**Table 1:** Antimicrobial agents tested for the different species analyzed in this study and criteria applied for categorizing the antimicrobial susceptibility profile.

Antimicrobial agent	Microorganism				Criteria
	*Staphylococcus* spp.	*E. faecalis*	*Enterobacterales*	*Pseudomonas spp* and other GNB	
Delafloxacin	X	X	X	X	X^a^
Ciprofloxacin	X	X	X	X	X^b^
Levofloxacin	X	X	X	X	X^b^
Tetracycline	X				X^b^
Linezolid	X	X			X^b^
Teicoplanin	X				X^b^
Vancomycin	X	X			X^b^
Oxacillin	X				X^b^
Cefepime			X	X	X^b^
Ceftazidime			X	X	X^b^
Imipenem			X	X	X^b^
Meropenem			X	X	X^b^
Ertapenem			X		X^b^
Amikacin			X	X	X^b^
Gentamicin			X	X	X^b^
Polymyxin B			X	X	X^b^

X^a^, FDA criteria.

X^b^, BRCAST criteria.

### Biofilm formation assay

The detection of biofilm formation was performed by microtiter plate assay, using crystal violet on a polystyrene abiotic surface. The results were interpreted as previously reported.^17^ First, the isolates were cultured in Tryptone Soy Broth (TSB) overnight, and then 5 µL of these cultures were inoculated in a 96-well-plate containing 195 µL of TSB in each well. The plate was incubated for 24 h at 37 °C. After the incubation, TSB was removed and the wells were washed three times with Phosphate Buffered Saline (PBS), fixed with formaldehyde 3 %, and stained with crystal violet 1 %. The dye was solubilized in ethanol 95 % and the Optical Density (OD) was read in a spectrophotometer with a wavelength of 570 nm. This assay was performed in triplicate.

### Detection of mutations in *gyrA* and *parC* in Gram-negative bacteria (GNB)

The delafloxacin-resistant GNB were selected to search for mutations in Quinolone Resistance Determining Regions (QRDR). The *gyrA* and *parC* genes were sequenced by Sanger method using specific primers (Table 2) for the selected isolates. Briefly, the amplicons were obtained by PCR and the DNA from PCR products were purified using the extraction kit Gel QIAquick (Qiagen, Courtaboeuf, France) according to manufacturer's instructions. The DNA quantification was performed in the NanoVue spectrophotometer (GE Healthcare, Canada) with a wavelength of 260 nm. For the sequencing, we used the Big Dye terminator Cycle Sequencing Kit (Applied Biosystems, Foster City, USA) and the run was performed in the ABI 3500 genetic Analyzer (Applied Biosystems, Perkin Elmer, USA) sequencer.

**Table 2:** Primers for *gyrA* and *parC* sequencing.

Primer	Sequence (5′−3′)	Target	Amplicon (bp)	Reference
*gyrA-F*	CGACCTTGCGAGAGAAAT			
		*gyrA*	626	Martins et al., 2015
*gyrA-R*	GTT CCATCAGCCCTTCAA			
				
*parC-F*	AGCGCCTTGCGTACATGA AT	*parC*	938	Martins et al., 2015
*parC-R*	GTGGTAGCGAAGAGGTGG TT			

The sequences obtained were analyzed in the Lasergene software (DNASTAR, Madison, USA) and the mutations analysis were performed using BioEdit® and SnapGene® software.

For evaluation of *gyrA* and *parC* mutations, we used different isolates’ sequences deposited in NCBI as controls: *E. coli* (NC_000913.3), *Klebsiella pneumoniae* (KN046818.1), *Pseudomonas aeruginosa* (NC_002516.2), *Enterobacter* spp. (NZ_MKEQ01000001.1), and *Morganella morganii* (NZ_JACOMH010000006.1).

### Detection of mecA gene

The *mecA* gene was searched in all *Staphylococcus* spp*.* isolates (*n* = 36) by PCR, using specific primers (*mecA*147-F: 5′-GTGAAGATATACCAAGTGATT-3′; *mecA*147-R: 5′-ATGCGCTATAGATTGAAAGGAT-3′) . The PCR conditions were as follows: 94 °C for 5 min, 30 cycles at 94 °C for 1 min, 55 °C for 1 min, 72 °C for 2 min, and the final extension at 72 °C for 10 min. ^18^

## Results

### Isolates characterization

Between January and April 2021, we collected 100 isolates recovered from 77 in patients diagnosed with ABSSI or osteomyelitis. Among the isolates, 58 % were GNB and 42 % were Gram-positive cocci.

The Enterobacterales corresponded to 63.8 % of the GNB with higher frequency of *Klebsiella pneumoniae*, followed by the non-fermenting GNB (36.2 %) with higher frequency of *Pseudomonas aeruginosa*. Among the Gram-positive bacteria, the most common genus was *Staphylococcus* spp. (*n* = 36/42), from which 50 % were identified as *S. aureus* and the other 50 % as belonging to the coagulase-negative group, represented by *S. epidermidis* (*n* = 10), *S. capitis* (*n* = 4), *S. hominis* (*n* = 2), *S. haemolyticus* (*n* = 1), and *S. warnerii* (*n* = 1).

**Fig. 1:**
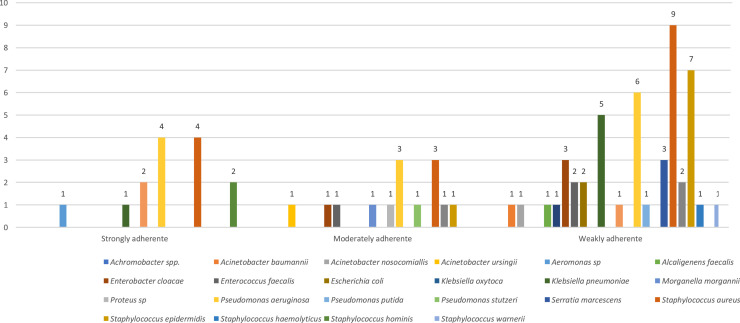
Species distribution of biofilm producers.

Overall, the most frequent pathogenic species obtained were *Staphylococcus aureus* (*n* = 18), followed by *Pseudomonas aeruginosa* (*n* = 14), *Klebsiella pneumoniae* (*n* = 9), and *Enterobacter cloacae* (*n* = 7) (Fig. 1). The microorganisms were isolated mostly from skin injuries (*n* = 58) and bone tissue (*n* = 13) from 77 patients. From these, 59 presented monomicrobial infections and 18 polymicrobial infections (two [*n* = 15] and three [*n* = 3] pathogens). The isolates were recovered from patients often hospitalized in the emergency room and surgery center.

### Antimicrobial susceptibility testing

In general, we observed a delafloxacin MIC ranging from ≤ 0.008 to > 4 mg/L, and the delafloxacin susceptibility rate was an average of 72.7 %.

*S. aureus* presented a susceptibility rate of 83.4 % to delafloxacin, with MIC_50/90_ of ≤ 0.008 and 2 mg/L, respectively. For the other comparators, the susceptibilities ranged from 27.8 % for tetracycline to 100 % for vancomycin and teicoplanin. According to the oxacillin susceptibility profile, nine *S. aureus* were classified as Methicillin-Resistant (MRSA) and nine were classified as Methicillin-Susceptible (MSSA). All the MSSA (100 %) were susceptible to delafloxacin (MIC_50_ ≤ 0.008 mg/L) and presented lower susceptibility rates for levofloxacin (11.1 % MIC_50_ 0.5 mg/L), ciprofloxacin (77.8 % ‘susceptible, increasing the exposure’; MIC_50_ 1 mg/L), and tetracycline (11.1 %; MIC_50_ 2 mg/L). For the MRSA, the delafloxacin susceptibility rate was 66.7 % (MIC_50_ ≤ 0.008 mg/L), which was higher than the susceptibility obtained for the fluoroquinolone comparators [levofloxacin and ciprofloxacin (66.7 % ‘susceptible, increasing the exposure’; MIC_50_ 0.5/1 mg/L)].

Among the CoNS, the susceptibility rate of delafloxacin was 83.3 % (MIC_50/90_ 0.06/1 mg/L). This was higher than that for levofloxacin (44.4 % ‘susceptible, increasing the exposure’; MIC_50/90_ 4/ > 4 mg/L) and ciprofloxacin (38.9 % ‘susceptible, increasing the exposure’; MIC_50/90_ 4/ > 4 mg/L). The susceptibility for the other antimicrobials ranged from 33.3 % for tetracycline to 100 % for vancomycin and teicoplanin.

*P. aeruginosa* presented a delafloxacin susceptibility rate of 71.4 % (MIC_50/90_ 0.25/1 mg/L). For the other fluoroquinolones, the susceptibility rates were 50 % of ‘susceptible, increasing the exposure’ (MIC_50/90_ 0.5/ > 4 mg/L) for levofloxacin and 42.9 % ‘susceptible, increasing the exposure’ (MIC_50/90_ 1/ > 4 mg/L) for ciprofloxacin. All *P. aeruginosa* isolates presented susceptibility to polymyxin B and resistance to carbapenems greater than 40 %.

Delafloxacin susceptibility rate against *K. pneumoniae* was 30 % (MIC_50/90_ 1/ > 4 mg/L). For the other fluoroquinolone comparators, the susceptibility rates were 20 % for levofloxacin (MIC_50/90_ 2/ > 4 mg/L) and 10 % for ciprofloxacin (MIC_50/90_ 4/ > 4 mg/L). The lowest susceptibility rate obtained was for ciprofloxacin and the highest were for amikacin and polymyxin B (60 %).

For *E. cloacae*, the delafloxacin susceptibility rate was 85.7 % (MIC_50_ 0.03 mg/L) which was the same value obtained for ciprofloxacin (MIC_50_ 0.03 mg/L), and both were lower than that obtained for levofloxacin (100 %; MIC_50_ 0.12 mg/L). In general, for *E. cloacae,* the susceptibility rates were higher than 70 %, except for ceftazidime (42.9 %) and cefepime (57.1 %).

For the other Enterobacterales (*Citrobacter freundii* = 2*; Morganella morgannii* = 3*; E. coli* = 4*; Serratia marcescens* = 4*;* and *Proteus spp*.= 6), the MIC_50_ was 0.25 mg/L and the MIC_90_ was 4 mg/L. Moreover, for the other species encountered (one isolate per species), the MIC for *Achromobacter* spp. was 0.12 mg/L; for *Acinetobacter baumannii*, 0.25 mg/L; for *A. nosocomialis, A. ursingii*, and *Aeromonas* spp., the MIC was ≤0.008 mg/L each. The overall susceptibility rates and the MIC_50/90_ for the antimicrobial agents are shown in Table 3. The MIC frequency distributions for delafloxacin and fluoroquinolone comparators are presented in Table 4 for the most frequent species.

**Table 3:** Activity of delafloxacin and comparators against ABSSSI isolates from Brazilian samples.

Microorganism/Antimicrobial agent	MIC (mg/L)					
	MIC_50_	MIC_90_	MIC range	%S	%I	%R
***Staphylococcus aureus* (*n*=18)**						
Delafloxacin^e^	≤ 0.008	2	≤ 0.008 ‒ 2	83.4	‒	16.6
Levofloxacin	0.5	> 4	0.12 ‒ > 4	5.6	66.7	27.8
Ciprofloxacin	1	> 4	≤ 0.008 ‒ > 4	‒	72.2	27.8
Oxacillin^b^	4	> 16	≤ 0.5 ‒ > 16	50	‒	50
Vancomycin	1	2	1 ‒ 2	100	‒	‒
Teicoplanin	≤ 0.25	0.25	≤ 0.25 – 0.5	100	‒	‒
Linezolid	1	2	1 ‒ 4	100 %	‒	‒
Tetracycline	2	> 8	0.5 ‒ > 8	27.8	38.9	33.3
**MSSA (*n*=9)**						
Delafloxacin^e^	≤ 0.008	a	≤ 0.008‒4	100	‒	‒
Levofloxacin	0.5	a	0.12 ‒ > 4	11.1	66.7	22.1
Ciprofloxacin	1	a	≤ 0.008 ‒ > 4	‒	77.8	22.2
Oxacillin^b^	≤ 0.5	a	≤ 0.5 ‒ 2	100	‒	‒
Vancomycin	1	a	≤ 0.25 ‒ 2	100	‒	‒
Teicoplanin	≤ 0.25	a	≤ 0.25 ‒ 0.25	100	‒	‒
Linezolid	1	a	0.5 ‒ 4	100	‒	‒
Tetracycline	2	a	0.5 ‒ > 8	11.1	55.6	33.3
**MRSA (*n*=9)**						
Delafloxacin^e^	≤ 0.008	a	≤ 0.008 ‒ 2	66.7	‒	33.3
Levofloxacin	0.5	a	0.5 ‒ > 4	‒	66.7	33.3
Ciprofloxacin	1	a	0.5 ‒ > 4	‒	66.7	33.3
Oxacillin^b^	> 16	a	4 ‒ > 16	‒	‒	100
Vancomycin	1	a	1 ‒ 2	100	‒	‒
Teicoplanin	0.25	a	≤ 0.25 ‒ 0.25	100	‒	‒
Linezolid	1	a	1 ‒ 2	100	‒	‒
Tetracycline	2	a	1 ‒ > 8	44.5	22.2	33.3
CoNS (*n*=18)*						
Delafloxacin^c^	0.06	1	≤ 0.008 ‒ 4	83.3	5.6	11.1
Levofloxacin	4	> 4	0.25 ‒ > 4	‒	44.4	55.6
Ciprofloxacin	4	> 4	0.12 ‒ > 4	‒	38.9	61.1
Oxacillin^b^	16	> 16	≤ 0.5 ‒ > 16	‒	‒	100
Vancomycin	2	4	1 ‒ 4	100	‒	‒
Teicoplanin	1	2	≤ 0.25 ‒ 2	100	‒	‒
Linezolid	0.5	4	0.25 ‒ 4	100	‒	‒
Tetracycline	4	8	1 ‒ > 8	33.3	5.6	61.1
***Klebsiella* spp. (*n*=10)**						
Delafloxacin^e^	1	> 4	≤ 0008 ‒ > 4	30	‒	70
Levofloxacin	2	> 4	≤ 0.008 ‒ > 4	20	10	70
Ciprofloxacin	4	> 4	≤ 0.008 ‒ > 4	10	10	80
Cefepime	64	> 64	≤ 0.12 ‒ > 64	33.3	‒	77.7
Ceftazidime	64	> 64	0.25 ‒ > 64	40	‒	60
Imipenem	1	64	0.25 ‒ 64	50	‒	50
Meropenem	4	32	≤ 0.12 ‒ > 64	40	10	50
Ertapenem	0.5	> 64	≤ 0.12 ‒ > 64	20	‒	80
Amikacin	2	> 64	1 ‒ > 64	60	‒	40
Gentamicin	32	> 64	0.25 ‒ > 64	20	‒	80
Polymyxin B	≤ 0,25	32	≤ 0.25 ‒ 64	60	‒	40
***Klebsiella pneumoniae***^d^**(*n*=9)**						
Delafloxacin^e^	1	a	0.06 ‒ > 4	22.2	‒	77.8
Levofloxacin	4	a	0.25 ‒ > 4	11.1	11.1	77.8
Ciprofloxacin	> 4	a	0.5 ‒ > 4	‒	11.1	88.9
Cefepime	> 64	a	≤ 0.12 ‒ > 64	33.3	‒	77.7
Ceftazidime	64	a	0.25 ‒ > 64	33.3	‒	77.7
Imipenem	32	a	0.25 ‒ 64	44.5	‒	55.5
Meropenem	32	a	≤ 0.12 ‒ > 64	33.3	11.1	55.5
Ertapenem	64	a	≤ 0.12 ‒ > 64	11.1	‒	88.9
Amikacin	2	a	1 ‒ > 64	55.5	‒	44.4
Gentamicin	32	a	0.25 ‒ > 64	11.1	‒	88.9
Polymyxin B	0.25	a	≤ 0.25 ‒ 64	55.5	‒	44.4
***Enterobacter cloacae* (*n*=7)**						
Delafloxacin^e^	0.03	a	≤ 0.008 ‒ 1	85.7	‒	14.3
Levofloxacin	0.12	a	0.03 ‒ 0.25	100	‒	‒
Ciprofloxacin	0.03	a	≤ 0.008 ‒ 0.5	85.7	14.3	‒
Cefepime	1	a	≤ 0.12 ‒ > 64	57.1	14.3	28.6
Ceftazidime	4	a	0.5 ‒ > 64	42.9	14.3	42.9
Imipenem	1	a	0.25 ‒ 4	71.4	28.6	‒
Meropenem	≤ 0.12	a	≤ 0.12 ‒ 4	71.4	28.6	‒
Ertapenem	≤ 0.12	a	≤ 0.12 ‒ 32	71.4	‒	28.6
Amikacin	2	a	0.25 ‒ > 64	85.7	‒	14.3
Gentamicin	0.25	a	≤ 0.12 ‒ 64	71.4	‒	28.6
Polymyxin B	≤ 0.25	a	≤ 0.25 ‒ > 128	71.4	‒	28.6
***Pseudomonas* spp*.***^f^**(*n*=16)**						
Delafloxacin^e^	0.25	1	0.016 ‒ > 4	81.3	12.5	6.2
Levofloxacin	0.5	> 4	0.03 ‒ > 4	‒	50	50
Ciprofloxacin	1	> 4	0.016 ‒ > 4	‒	37.5	62.5
Cefepime	4	> 64	≤ 0.12 ‒ > 64	‒	50	50
Ceftazidime	8	32	0.25 ‒ 64	‒	87.5	12.5
Imipenem	4	16	0.25 ‒ 16	‒	43.8	56.2
Meropenem	8	32	0.25 ‒ 64	43.8	18.7	37.5
Amikacin	4	> 64	0.5 ‒ > 64	68.7	‒	31.3
Gentamicin^g^	2	> 64	≤ 0.12 ‒ > 64	–	‒	‒
Polymyxin B	0.5	1	≤ 0.25 ‒ 8	93.7	‒	6.3
***Pseudomonas aeruginosa* (*n*=14)**						
Delafloxacin^e^	0.25	1	0.016 ‒ > 4	78.7	14.2	7.1
Levofloxacin	0.5	> 4	0.03 ‒ > 4	‒	50	50
Ciprofloxacin	1	> 4	0.016 ‒ > 4	‒	42.9	57.1
Cefepime	16	> 64	1 ‒ > 64	‒	42.9	57.1
Ceftazidime	4	32	0.25 ‒ 64	‒	85.7	14.3
Imipenem	4	16	1 ‒ 16	‒	50	50
Meropenem	8	32	0.25 ‒ 64	42.9	14.3	42.9
Amikacin	8	> 64	2 ‒ > 64	64.3	‒	35.7
Gentamicin	4	> 64	1 ‒ > 64	g	g	g
Polymyxin B	0.5	1	≤ 0.25 ‒ 1	100	‒	‒

⁎ All CoNS were resistant to oxacillin.

a It was not possible to calculate the MIC_90_ because the isolates number was lower than 10.

b Categorization performed according to BRCAST/EUCAST (2021): *S. aureus* isolates presenting MIC > 2 mg/L for oxacillin were categorized as resistant to methicillin.

c All CoNS were classified for delafloxacin according to the breakpoint for *S. haemolyticus,* preconized by the FDA (2020).

d *Klebsiella spp, Klebsiella oxytoca* (1) and *Klebsiella pneumoniae* (9).

e AST categorization for delafloxacin according to the breakpoints preconized by the FDA (2020). For the comparators the BRCAST/EUCAST (2021) breakpoint were used.

f *Pseudomonas* spp. *Pseudomonas aeruginosa* (14), *Pseudomonas putida* (1) and 1 *Pseudomonas stutzeri* (1).

g There is no breakpoint established by BRCAST/EUCAST (2021).

**Table 4:** Delafloxacin and quinolone comparators MIC frequency distributions for the most frequent ABSSSI isolates.

	N° (cumulative %) of isolates inhibited at MIC (mg/L) of:										
Microorganism or Microorganism group/ Antimicrobial agent	≤ 0.008	0.016	0.03	0.06	0.12	0.25	0.5	1	2	≥ 4	n (R%)
***S. aureus* (*n*=18)**											
Delafloxacin^a^	11 (61.1 %)	1 (66.7 %)	0	1 (72.2 %)	1 (77.8 %)	1 (83.3 %)	0	0	2 (94.4)	1 (100 %)	3 (16.6)
Levofloxacin	0	0	0	0	1 (5.6 %)	0	12 (72.2 %)	0	0	5 (100 %)	5 (27.8)
Ciprofloxacin	1 (5.6 %)	0	0	0	0	0	3 (22.2 %)	9 (72.2 %)	0	5 (100 %)	5 (27.8)
***Staphylococcus* Coagulase Negative (*n*=18)**											
Delafloxacin^a^	6 (33.3 %)	1 (38.9 %)	1 (44.5 %)	2 (55.7 %)	2 (66.9 %)	3 (83.7 %)	1 (89.3 %)	1 (94.9 %)	0	1 (100 %)	2 (11.1)
Levofloxacin	0	0	0	0	0	2 (11.1 %)	6 (44.4 %)	0	0	10 (100 %)	10 (55.6)
Ciprofloxacin	0	0	0	0	1 (5.6 %)	5 (33.3 %)	0	1 (38.9 %)	0	11 (100 %)	11 (61.1)
***Enterobacter cloacae* (*n*=7)**											
Delafloxacin^b^	2 (28.6 %)	1 (42.9 %)	2 (7.,4 %)	0	0	0	1 (85.7 %)	1 (100 %)	0	0	1 (14.3)
Levofloxacin			2 (28.6 %)	1 (42.9 %)	3 (85.7 %)	1 (100 %)	0	0	0	0	0
Ciprofloxacin	3 (42.9 %)	0	1 (57.1 %)	2 (85.7 %)	0	0	1 (100 %)	0	0	0	0
***Pseudomonas* spp. (*n*=16)**											
Delafloxacin^b^		1 (6.3 %)	3 (25.0 %)	2 (37.5 %)	1 (43.8 %)	5 (75.0 %)	1 (81.3 %)	2 93.8 %)	0	1 (100 %)	1 (6.2)
Levofloxacin	0	0	1 (6.3 %)	1 (12.5 %)	0	4 (37.5 %)	2 (50.0 %)	0	4 (75.0 %)	4 (100 %)	8 (50.0)
Ciprofloxacin	0	2 (12.5 %)	0	3 (31.3 %)	0	1 (37.5 %)	0	2 (50.0 %)	2 (62.5 %)	6 (100 %)	10 (62.5)
***Klebsiella* spp. (*n*=10)**											
Delafloxacin^b^	1 (10.0 %)	0	0	1 (20.0 %)	0	1 (30.0 %)	0	3 (60.0 %)	2 (80.0 %)	2 (100 %)	7 (70.0)
Levofloxacin	1 (10.0 %)	0	0	0	0	1 (20.0 %)	0	1 (30.0 %)	2 (50.0 %)	5 (100 %)	7 (70.0)
Ciprofloxacin	1 (10.0 %)	0	0	0	0	0	1 (20.0 %)	0	0	8 (100 %)	8 (80.0)

Shaded cells indicate the breakpoints for each antimicrobial agent according to BRCAST/EUCAST (2021) or FDA (2020).

a All CoNS were classified for delafloxacin according to the breakpoint for *S. haemolyticus,* preconized by the FDA (2020).

b Delafloxacin breakpoints used are from FDA (2020) and for the other quinolone comparators breakpoints are from BRCAST/EUCAST (2021).

### Biofilm formation assay

Among the 100 isolates, 25 % were categorized as non-adherent, and 75 % were categorized as biofilm producers, with 47 % being classified as weakly adherent, 14 % as moderately adherent, and 14 % as strongly adherent.

The most common species of biofilm producers were *P. aeruginosa, S. aureus*, and *S. epidermidis.* The moderately and strongly adherent isolates were mostly *P. aeruginosa* (*n* = 3 and *n* = 4) and *S. aureus* (*n* = 3 and *n* = 4) (Fig. 1).

Moreover, we observed a good activity of delafloxacin against different biofilm-producing isolates (*S. aureus, Enterococcus faecalis, P. aeruginosa, E. cloacae, Proteus* spp., and CoNS). Among the biofilm-producers, those strongly and moderately adherent (28/75) presented a MIC range of ≤ 0.008 mg/L to > 4 mg/L, and the majority (23/28) presented MIC ≤ 0.25 mg/L. The strongly adherent isolates presented a delafloxacin susceptibility rate of 71.4 % and the moderately adherent 85.7 %.

### Detection of mutations in QRDR of Gram-negative bacteria

Among 58 GNBs, 17 were resistant to delafloxacin. From these, 13 presented mutations in *parC* and 14 presented mutations in *gyrA.* In ParC protein, the predominant amino acid alteration was observed in position 80, where a serine was replaced by an Isoleucine (S80I) in *E. coli* and *K. pneumoniae* species. Also, we observed D79Y, A81P, and N105I mutations in *K. pneumoniae*, a deletion at position 21 and a substitution at position 87 (S87L) in *P. aeruginosa.* In GyrA protein, amino acid changes were more frequent at position 83. In *E. coli*, we detected S83L; in *P. aeruginosa*, T83I; and in *K. pneumoniae*, S83I and S83F. Moreover, we observed changes at position 87 (*E. coli*, D87N; and *K. pneumoniae*, D87A) and a deletion at position 163 in *P. aeruginosa.*

### *mecA* gene detection in *Staphylococcus* spp

Among the 36 *Staphylococcus* spp. isolates (18 *S. aureus* and 18 CoNS), the *mecA* gene was detected in 77.7 % (*n* = 28/36). For *S. aureus*, 61.1 % (*n* = 11/18) were *mecA*-positive while 94.4 % (*n* = 17/18) were *mecA* positive for CoNS.

We could observe that among the 11 *mecA-*positive *S. aureus*, nine presented a resistance phenotype to oxacillin (MIC > 2 mg/L). Also, among the 18 oxacillin-resistant CoNS (MIC > 0.25 mg/L), 17 were *mecA-*positive.

## Discussion

The new fluoroquinolone, delafloxacin, was approved for ABSSSI treatment and is active against Gram-negative and Gram-positive pathogens, including *S. aureus* (MSSA and MRSA), CoNS (*S. haemolyticus* and *S. lugdunensis*), *Streptococcus* spp*., Enterococcus faecalis, E. coli, E. cloacae, K. pneumoniae* and *P. aeruginosa*.^13^^,^^19^ Also, the FDA has approved its use for the treatment of community-acquired pneumonia.^3^ There are some publications showing good outcomes of delafloxacin use in clinical practice.^20^, ^21^, ^22^ Delafloxacin was successfully employed for treatment of eight patients with complicated ABSSSI admitted to Brazilian public teaching and reference hospital in infectious diseases from October 2022 to April 2023. Delafloxacin showed to be safe and effective for treating complicated ABSSSI including those caused by MRSA in people living with HIV/AIDS.^23^

In the present study, we observed that delafloxacin presented an excellent activity against *S. aureus* (MIC_50_ ≤ 0.008 mg/L) and CoNS (MIC_50_ 0.06 mg/L) isolates, being at least 64 times more potent than both levofloxacin and ciprofloxacin (*S. aureus*; MIC_50_ 0.5 mg/L; and CoNS; MIC_50_ 4 mg/L). Overall, for *Staphylococcus* spp., delafloxacin was more active than the other fluoroquinolones comparators (Table 4). McCurdy and collaborators also obtained high rates of delafloxacin activity against levofloxacin-resistant *S. aureus*, with 95.0 % susceptibility to delafloxacin.^24^ Another study conducted in Europe showed that 92.4 % *S. aureus* were susceptible to delafloxacin (MIC_50/90_ ≤ 0.004/0.25), being more active than levofloxacin and moxifloxacin.^13^ Gerges and colleagues found delafloxacin susceptibilities of 40 % against MRSA, 80 % against MSSA, 50 % against methicillin-resistant-resistant CoNS and 95 % against methicillin-susceptible CoNS in pathogens recovered from oncologic patients.^12^ In a Brazilian study, Barth and collaborators accessed a rate of 100 % of susceptibility to delafloxacin in *S. aureus* isolated from ABSSSI.^25^ Moreover, Nicola and colleagues found delafloxacin susceptibilities of 97.5 % against MRSA, 97.7 % against MSSA, 93.5 % against CoNS in pathogens recovered from osteoarticular and skin infections.^14^

Delafloxacin (MIC_50_ 0.25 mg/L) was at least four times more potent than ciprofloxacin (MIC_50_ 1 mg/L) against *P. aeruginosa*, with an inhibition rate of 71.4 %. We also observed that these isolates presented resistance rates to carbapenems ≥ 50 %. Millar and collaborators observed that 50 % of ciprofloxacin-resistant or ciprofloxacin-‘susceptible increasing the exposure’ *P. aeruginosa* isolated from cystic fibrosis infection were susceptible to delafloxacin.^26^ Recently, a study conducted in the USA showed a delafloxacin susceptibility rate of 40 % in *P. aeruginosa*, with a rate of 75 % in *P. aeruginosa* non-MDR.^13^ Although all the *P. aeruginosa* isolates in this study were susceptible (100 %) to polymyxin, it is important to highlight that this drug presents high toxicity.^27^ Recently, another study conducted in the USA with isolates from ABSSSI, between 2017 and 2022, showed an overall susceptibility to delafloxacin of 70.3 %, with an increase of 8.8 % in the susceptibility rate.^28^

For *E. cloacae*, delafloxacin activity (MIC_50_ 0.03 mg/L) was equal to ciprofloxacin (MIC_50_ 0.03 mg/L) as well as the susceptibility rate (85.7 %). Similar results were obtained by Gerges and colleagues who observed a susceptibility rate of 85 % for these antimicrobials.^12^

Furthermore, in this study, delafloxacin presented a low activity against *K. pneumoniae* (22.2 %), as well as levofloxacin (11.1 %) and ciprofloxacin (11.1 %, ‘susceptible, increasing the exposure’). This could be explained by the high frequency of MDR*-K. pneumoniae* in the involved hospital, especially to aminoglycosides, carbapenems and polymyxin B^29^ as noted in Table 3. Another study showed 70 % of susceptibility to delafloxacin in *K. pneumoniae*, but these isolates were classified as non-ESBL and were susceptible to carbapenem.^12^

Moreover, we observed a good activity of delafloxacin against different biofilm-producing isolates. Interestingly, among these isolates, the majority (23/28) presented delafloxacin MIC ≤ 0.25 mg/L and the strongly adherent isolates presented a delafloxacin susceptibility rate of 71.4 % and the moderately adherent, 85.7 %. As it is already known, fluoroquinolones display good efficacy in treating osteomyelitis, due to their action on biofilm.^30^^,^^31^ Although clinical studies on the use of delafloxacin for osteomyelitis are scarce,^32^ recently a study of case was reported and a sacral osteomyelitis caused by *P. aeruginosa* that was not resolved after using polymyxin followed by ceftazidime/avibactam, was then extinguished after endovenous administration of delafloxacin.^33^ Previous studies had shown a potent activity of delafloxacin against biofilms from *S. aureus*, thus presenting an antimicrobial penetration from 0.6 % to 52 % on biofilm.^34^^,^^35^ In the present study, we did not test the activity of delafloxacin against biofilm, but against biofilm-producing isolates, hypothesizing that the antimicrobial could act against these isolates even before their biofilm formation.

Furthermore, mutations in *gyrA* and *parC* genes are recognized to be the main mechanism of resistance which confer a high-level resistance to fluoroquinolones. These mutations can confer amino acid alterations in these proteins, reflecting fluoroquinolone resistance.^36^ In the present study, we found amino acid changes in GyrA from *E. coli, P. aeruginosa*, and *K. pneumoniae*. Mostly, the amino acid in position 83 was replaced in all these three species. Also, the D87N/A change was detected in *E. coli* and *K. pneumoniae*; and in *P. aeruginosa*, a deletion at position 163 was observed. The most common mutations in *gyrA* related to fluoroquinolones resistance are associated with positions 83 and 87.^37^^,^^38^ However, to the best of our knowledge, this is the first time that the deletion in position 163 of GyrA in *P. aeruginosa* is reported as possibly to be related to fluoroquinolone resistance.

Furthermore, for *parC* gene, we observed amino acid changes mostly in position 80 in *E. coli* and *K. pneumoniae,* 87 in *P. aeruginosa*, 79 and 81 in *K. pneumoniae*. Also, a deletion in position 27 in *P. aeruginosa* was observed. The S80I substitution is already recognized to be related to fluoroquinolone resistance, as well as S87L in *P. aeruginosa*.^39^^,^^40^ However, to date, the mutations (D79 and A81P) in *K. pneumoniae* and deletion at position 27 in *P. aeruginosa* have not been reported to be possibly associated with fluoroquinolone resistance.

Finally, we could observe that delafloxacin presented a good activity against the *Staphylococcus* spp*.* resistant to oxacillin, with delafloxacin-susceptible MRSA rate of 66.7 % and delafloxacin-susceptible CoNS rate of 83.3 %. We also observed that 82.1 % of the *Staphylococcus* spp. harboring *mecA* gene were susceptible to delafloxacin. The study conducted by Saravolatz and collaborators assessed oxacillin susceptibility based on SCC*mec* typing for MRSA and showed that delafloxacin demonstrated activity against 94 % of SCC*mec* IVa USA300 isolates.^41^ On the other hand, our study is the first to present delafloxacin activity against isolates harboring the *mecA* gene.

However, our study shows limitations. The principal limitation of our work is the low number of isolates analyzed based on species. As we had a wide variety of species, the selected 100 isolates were distributed among them, thereby reflecting a low number by species. It is also important to highlight that we tested delafloxacin activity against biofilm producing isolates and not against the produced biofilm. Further studies are however needed to evaluate the activity of this drug on biofilm.

## Conclusions

In the present study, we conducted a comparative analysis of delafloxacin's in vitro activity with other antimicrobials against various bacterial isolates obtained from patients diagnosed with ABSSSI or osteomyelitis. Among the fluoroquinolones, delafloxacin exhibited superior activity against the isolates, demonstrating up to 64 times greater potency than levofloxacin and ciprofloxacin. Furthermore, our findings revealed that delafloxacin displayed notable efficacy against MRSA, MSSA, CoNS and *P. aeruginosa* strains isolated in Brazil.

The *gyrA* and *parC* genes sequencing results revealed that there are different amino acid substitutions and deletions which might be related to fluoroquinolone resistance, thus highlighting the need for more studies to evaluate the impact of these mutations.

Interestingly, we observed a good activity of delafloxacin against biofilm-producing isolates, presuming that this antimicrobial could act against bacteria even before the formation of biofilm.

## Conflicts of interest

The authors declare no conflicts of interest.
